# Influence of Traps and Lorentz Force on Charge Transport in Organic Semiconductors

**DOI:** 10.3390/ma16134691

**Published:** 2023-06-29

**Authors:** Seema Morab, Manickam Minakshi Sundaram, Almantas Pivrikas

**Affiliations:** College of Science, Health, Engineering and Education, Murdoch University, Perth, WA 6150, Australia

**Keywords:** Hall effect, organic semiconductors, traps, Lorentz force, Langevin recombination, charge transport

## Abstract

Charge transport characteristics in organic semiconductor devices become altered in the presence of traps due to defects or impurities in the semiconductors. These traps can lead to a decrease in charge carrier mobility and an increase in recombination rates, thereby ultimately affecting the overall performance of the device. It is therefore important to understand and mitigate the impact of traps on organic semiconductor devices. In this contribution, the influence of the capture and release times of trap states, recombination rates, and the Lorentz force on the net charge of a low-mobility organic semiconductor was determined using the finite element method (FEM) and Hall effect method through numerical simulations. The findings suggest that increasing magnetic fields had a lesser impact on net charge at constant capture and release times of trap states. On the other hand, by increasing the capture time of trap states at a constant magnetic field and fixed release time, the net charge extracted from the semiconductor device increased with increasing capture time. Moreover, the net charge extracted from the semiconductor device was nearly four and eight times greater in the case of the non-Langevin recombination rates of 0.01 and 0.001, respectively, when compared to the Langevin rate. These results imply that the non-Langevin recombination rate can significantly enhance the performance of semiconductor devices, particularly in applications that require efficient charge extraction. These findings pave the way for the development of more efficient and cost-effective electronic devices with improved charge transport properties and higher power conversion efficiencies, thus further opening up new avenues for research and innovation in this area of modern semiconductor technology.

## 1. Introduction

Organic semiconductors have shown great potential in applications such as flexible electronics, solar cells [1], and light-emitting diodes [2]. These materials are composed of carbon-based molecules and offer advantages over traditional inorganic semiconductors, such as low-cost processing and the ability to be printed onto various substrates. Additionally, organic semiconductors have the potential for high performance in electronic devices due to their tuneable energy levels and charge transport properties. However, challenges still exist in improving the stability and efficiency of these materials. Researchers are actively working on developing new organic semiconductors with improved properties as well as optimising device architectures to maximise performance. Additionally, advances in computational modelling and simulation are helping to accelerate the discovery and design of new materials with desirable properties. As these efforts continue, we may see even more exciting applications emerge for these versatile materials in the future.

The charge transport in organic semiconductors plays a vital role in device efficiency and performance. By tailoring the molecular structure of these materials, researchers can improve their charge mobility and reduce the likelihood of energy loss through non-radiative recombination. One approach to enhancing the charge transport in organic semiconductors is through the use of dopants, which can introduce additional charge carriers and improve conductivity [3]. Another strategy involves optimising the morphology of the material, such as by controlling the orientation and packing of molecules to minimise disorder and enhance charge transport pathways [4]. Additionally, incorporating interfacial layers between the organic semiconductor and other materials in a device can help improve charge injection and extraction [5]. Furthermore, advancements in mathematical modelling and simulation techniques can enable a better understanding of the fundamental mechanisms underlying charge transport in these materials, thereby allowing for more informed design choices. As organic electronics continue to gain traction in various applications, further improvements in the charge transport will be critical for achieving higher performance and efficiency.

The charge transport properties in organic semiconductors are altered by the presence of traps and defects, which can limit the efficiency and performance of electronic devices. For example, in the case of organic light-emitting diodes (OLEDs), charge carriers such as electrons and holes emanating from opposite electrodes recombine through radiative recombination to produce light. However, due to the presence of traps in OLEDs, non-radiative recombination occurs between the charge carriers, thereby lowering the device’s efficiency [6,7,8]. Moreover, this impact of traps leads to the degradation of the device by decreasing the mobility of the charge carriers [9,10]. In the case of organic photovoltaics (OPVs), light absorption from two separate organic semiconductors produces electron-hole pairs. These electron–hole pairs decompose at the organic–organic heterojunction into free charge carriers and are moved to an external circuit separately. In the presence of traps, these separated charge carriers recombine through non-radiative Shockley–Read–Hall (SRH) recombination, thereby lowering the device’s efficiency [11,12]. Additionally, the alignment of the energy levels at the heterojunction is also altered by the traps [13,14,15]. Conversely, electron–hole pairs can be decomposed into free charge carriers with the help of traps [16]. These traps can help overcome the high Coulomb energy barrier binding the electron–hole pairs to decompose into free charge carriers, such that free charges move to lower energy states created by traps after decomposition. This process increases the device’s efficiency.

Traps usually occur because of the presence of defects or impurities in semiconductors. These defects can cause disruptions in the flow of electrons, thus leading to unintended consequences. In addition to defects, impurities such as dopants can also create traps in semiconductors. These impurities can alter the electronic properties of the material and create regions with different conductivity or charge carrier concentrations. Overall, understanding and controlling traps is crucial for optimising semiconductor performance and reliability. This work aims to provide information on the influence of traps, Langevin and non-Langevin recombination rates, and the Lorentz force on the net charge of a low-mobility organic semiconductor device in two dimensions using the Hall effect method and the finite element method (FEM). Starting with the methodology for device modelling and simulations in Section 2, the effect of traps and the implications of the magnetic field on the net charge of the organic semiconductor device are presented in Section 3. The conclusion is discussed in Section 4.

## 2. Methodology for Device Modelling and Simulations

A one-dimensional drift–diffusion–recombination solver was used to develop a one-dimensional thin film semiconductor device model [17,18,19,20,21,22]. Furthermore, this model was expanded to two dimensions to implement the Lorentz force on the device. External electric and magnetic fields were applied to the semiconductor, and two electrodes injected the charge carriers (electrons and holes). Eight trap states were incorporated into the semiconductor without dopants. Capture and release times were specified to identify the presence of charge carriers in these trap states. Poisson and continuity equations were applied to the charge carriers within the semiconductor [17,18,19,23,24]. A simple trapping model was employed [25]. The equations used in this model were all normalised by adopting the technique analogous to Juška et al. [26,27], and more information on normalisation can be found here [28]. A prime symbol was used to denote the non-dimensionalized quantities. The trap model equations that were normalised are shown below.

(1) $$\frac{\partial n^{\prime }\left(x^{\prime },t^{\prime }\right)}{\partial t^{\prime }}-\frac{\partial j_{n}^{\prime }\left(x^{\prime },t^{\prime }\right)}{\partial x^{\prime }}=g^{\prime }\left(x^{\prime },t^{\prime }\right)-r_{FF}^{\prime }\left(x^{\prime },t^{\prime }\right)-r_{FT}^{\prime }\left(x^{\prime },t^{\prime }\right)-r_{TF}^{\prime }\left(x^{\prime },t^{\prime }\right)$$

(2) $$\frac{\partial n^{\prime }\left(y^{\prime },t^{\prime }\right)}{\partial t^{\prime }}-\frac{\partial j_{n}^{\prime }\left(y^{\prime },t^{\prime }\right)}{\partial y^{\prime }}=g^{\prime }\left(y^{\prime },t^{\prime }\right)-r_{FF}^{\prime }\left(y^{\prime },t^{\prime }\right)-r_{FT}^{\prime }\left(y^{\prime },t^{\prime }\right)-r_{TF}^{\prime }\left(y^{\prime },t^{\prime }\right)$$

(3) $$\frac{\partial p^{\prime }\left(x^{\prime },t^{\prime }\right)}{\partial t^{\prime }}+\frac{\partial j_{p}^{\prime }\left(x^{\prime },t^{\prime }\right)}{\partial x^{\prime }}=g^{\prime }\left(x^{\prime },t^{\prime }\right)-r_{FF}^{\prime }\left(x^{\prime },t^{\prime }\right)-r_{FT}^{\prime }\left(x^{\prime },t^{\prime }\right)-r_{TF}^{\prime }\left(x^{\prime },t^{\prime }\right)$$

(4) $$\frac{\partial p^{\prime }\left(y^{\prime },t^{\prime }\right)}{\partial t^{\prime }}+\frac{\partial j_{p}^{\prime }\left(y^{\prime },t^{\prime }\right)}{\partial y^{\prime }}=g^{\prime }\left(y^{\prime },t^{\prime }\right)-r_{FF}^{\prime }\left(y^{\prime },t^{\prime }\right)-r_{FT}^{\prime }\left(y^{\prime },t^{\prime }\right)-r_{TF}^{\prime }\left(y^{\prime },t^{\prime }\right)$$

(5) $$r_{FF}^{\prime }\left(x^{\prime },t^{\prime }\right)=r_{FF}^{\prime }\left(y^{\prime },t^{\prime }\right)=\beta_{ff}^{\prime }\left(\mu_{n}^{\prime }+\mu_{p}^{\prime }\right)\;n_{f}^{\prime }\;p_{f}^{\prime }$$

(6) $$r_{FT}^{\prime }\left(x^{\prime },t^{\prime }\right)=r_{FT}^{\prime }\left(y^{\prime },t^{\prime }\right)=\beta_{ft}^{\prime }\left(\mu_{n}^{\prime }+\mu_{p}^{\prime }\right)\;n_{f}^{\prime }\;p_{t}^{\prime }$$

(7) $$r_{TF}^{\prime }\left(x^{\prime },t^{\prime }\right)=r_{TF}^{\prime }\left(y^{\prime },t^{\prime }\right)=\beta_{tf}^{\prime }\left(\mu_{n}^{\prime }+\mu_{p}^{\prime }\right)\;n_{t}^{\prime }\;p_{f}^{\prime }$$

(8) $$\frac{\partial n_{f}^{\prime }}{\partial t^{\prime }}=-\frac{n_{f}^{\prime }}{\tau_{C}^{\prime }}+\frac{n_{t}^{\prime }}{\tau_{R}^{\prime }}$$

(9) $$\frac{\partial p_{f}^{\prime }}{\partial t^{\prime }}=-\frac{p_{f}^{\prime }}{\tau_{C}^{\prime }}+\frac{p_{t}^{\prime }}{\tau_{R}^{\prime }}$$

(10) $$\frac{\partial n_{t}^{\prime }}{\partial t^{\prime }}=\frac{n_{f}^{\prime }}{\tau_{C}^{\prime }}-\frac{n_{t}^{\prime }}{\tau_{R}^{\prime }}$$

(11) $$\frac{\partial p_{t}^{\prime }}{\partial t^{\prime }}=\frac{p_{f}^{\prime }}{\tau_{C}^{\prime }}-\frac{p_{t}^{\prime }}{\tau_{R}^{\prime }}$$

(12) $$\frac{\partial^{2}V^{{}^{\prime }}}{\partial {\left(x^{\prime }\right)}^{2}}=\left(n_{f}^{\prime }+n_{t}^{\prime }\right)-\left(p_{f}^{\prime }+p_{t}^{\prime }\right)$$

(13) $$\frac{\partial^{2}V^{{}^{\prime }}}{\partial {\left(y^{\prime }\right)}^{2}}=\left(n_{f}^{\prime }+n_{t}^{\prime }\right)-\left(p_{f}^{\prime }+p_{t}^{\prime }\right).$$

Equations (1) to (4) represent continuity equations for the number density of electrons ($n^{\prime }$) and holes ($p^{\prime }$) with generation and recombination rates of charge carriers denoted by $g^{\prime }$ and $r^{\prime }$, respectively, in both x and y directions. The generation rate was set to zero, and recombination rates ($r^{\prime }$) were defined by Equations (5) to (7), which demonstrate three possible recombinations between charge carriers. These included the recombination between free electrons ($n_{f}^{\prime }$) and free holes ($p_{f}^{\prime }$), free electrons ($n_{f}^{\prime }$) and trapped holes ($p_{t}^{\prime }$), and trapped electrons ($n_{t}^{\prime }$) and free holes ($p_{f}^{\prime }$). The recombination coefficient $\beta^{\prime }$ = 1 was defined for Langevin recombination, and $\beta^{\prime }<1$ was defined for non-Langevin recombination [29,30], with carrier mobilities denoted by $\mu^{\prime }=1$. Trapping equations are given by Equations (8) to (11) with capture and release times of trap states as $\tau_{C}^{\prime }$ and $\tau_{R}^{\prime }$, respectively, where $\tau_{C}^{\prime }$ = $\tau_{C_{n}}^{\prime }$ = $\tau_{C_{p}}^{\prime }$, and $\tau_{R}^{\prime }$ = $\tau_{R_{n}}^{\prime }$ = $\tau_{R_{p}}^{\prime }$. Equations (12) and (13) are Poisson equations.

All these model equations were incorporated into the COMSOL Multiphysics Software 6.0 version for computation to determine the net charge of the semiconductor device. The software performed the computation in five consecutive steps. These included: 1. Defining geometry; 2. Problem specification; 3. Meshing; 4. Solution; and 5. Post-processing and visualisation. In the first step, a two-dimensional geometry of the device with x and y values ranging from 0 to 1 was created. In the second step, all normalised model equations were incorporated into the model builder to define the problem. The problem was focused on the extraction of net charge from the semiconductor device. In the model builder, component 1 was selected, and then ‘Physics’ was added. Subsequently, mathematics and partial differential equation (PDE) interfaces were added to solve the partial differential equations, and then global parameters and variables were defined.

Seven general form PDEs were added to component 1, where the first and second PDEs solved the continuity equations for the electron ($n^{\prime }$) and hole ($p^{\prime }$) concentrations, respectively, given by Equations (1) to (4), with the conduction current density for electrons and holes given by $j_{n}^{\prime }\left(x^{\prime },t^{\prime }\right)=n^{\prime }\left(x^{\prime },t^{\prime }\right)E^{\prime }\left(x^{\prime },t^{\prime }\right)\mu {}_{n}^{\prime }+T^{\prime }\mu {}_{n}^{\prime }\frac{\partial n^{\prime }}{\partial x^{\prime }}$ and $j_{p}^{\prime }\left(x^{\prime },t^{\prime }\right)=p^{\prime }\left(x^{\prime },t^{\prime }\right)E^{\prime }\left(x^{\prime },t^{\prime }\right)\mu {}_{p}^{\prime }-T^{\prime }\mu {}_{p}^{\prime }\frac{\partial p^{\prime }}{\partial x^{\prime }}$ in the x direction. Normalised force $F^{\prime }\left(y^{\prime },t^{\prime }\right)=B^{\prime }\left(-z^{\prime },t^{\prime }\right)\;E^{\prime }\left(x^{\prime },t^{\prime }\right)\;\mu^{\prime }$ replaced electric field term in conduction current density equations for electrons and holes in the y direction. In these PDEs, the number of electrons and holes initially present at the injecting electrode was 100 at $x^{\prime }$ = 0 and $x^{\prime }$ = 1, respectively, which was given by the Dirichlet boundary condition, and fluxes 1 and 2 were defined by the drift component of $v_{d_{n}}=n^{\prime }\;E^{\prime }\;\mu {}_{n}^{\prime }$ or $v_{d_{p}}=p^{\prime }\;E^{\prime }\;\mu {}_{p}^{\prime }$ for electrons and holes, respectively. $n^{\prime }$, $p^{\prime }$, $\frac{\partial n^{\prime }}{\partial t^{\prime }}$, and $\frac{\partial p^{\prime }}{\partial t^{\prime }}$ had initial values of zero. The third PDE solved the Poisson equations given by Equations (12) and (13). In this PDE, voltage $V^{\prime }$ = 0 at $x^{\prime }$ = 0 and voltage $V^{\prime }$ = 1 at $x^{\prime }$ = 1 were applied in forward bias using the Dirichlet boundary condition. Furthermore, the temperature was fixed at $1\times 10^{-2}$, ‘Study’ was added to component 1, and a time-dependent case was selected to analyse the net charge of the semiconductor device. The fourth, fifth, sixth, and seventh PDEs solved the trapping Equations (8)–(11), respectively, with initial values of $n_{f}^{\prime }=0$, $p_{f}^{\prime }=0$, $n_{t}^{\prime }=if\left(n_{t}^{\prime }<N_{T},\tau_{C_{n}}^{\prime },infinity\right)$, $p_{t}^{\prime }=if\left(p_{t}^{\prime }<P_{T},\tau_{C_{p}}^{\prime },infinity\right)$, $\frac{\partial n_{t}^{\prime }}{\partial t^{\prime }}=0$, and $\frac{\partial p_{t}^{\prime }}{\partial t^{\prime }}=0$ in these PDEs. $N_{T}=4$ and $P_{T}=4$ represent electron and hole trap states, respectively, within the semiconductor domain.

In the third step, a mesh was added to component 1, and a user-controlled mesh with a free triangular distribution was selected. This distribution had a geometric entity level set to ‘remaining’ with a distribution type set to ‘fixed number of elements’, and the number of elements was 100. All boundaries were selected for the geometry, and the mesh size was kept at ‘fine’ for stability and convergence in results. In step four, the computation from ‘study 1’ was selected, and a colour surface plot appeared in the graphics window in which the colours visualised the net charge distribution within the semiconductor domain. In the final step, a one-dimensional plot was added from the model builder to the results section in software, and then a line graph was added to this plot.

## 3. Results and Discussion

The model developed in Section 2 for two-dimensional organic semiconductor devices was implemented in software for computation to measure the charge transport in semiconductors. Electrons and holes were injected on the left and right electrodes of the semiconductor, respectively, and external electric and magnetic fields were applied to the semiconductor. Trap states were incorporated into the semiconductor. Traps usually occur within the semiconductors as a consequence of defects and impurities present in the semiconductors. These trap states can significantly affect the charge transport properties of the semiconductor. The capture and release times were defined to determine the existence of charge carriers in these trap states. In the presence of traps, the overall charge concentration within the semiconductors decreases. However, with an increase in capture time, the total charge concentration increases. The results were obtained for the net charge extracted from the semiconductor device in the presence of Langevin- and non-Langevin-type recombinations between charge carriers, the Lorentz force, and traps. These results are discussed in Section 3.1 and Section 3.2.

### 3.1. Effect of Traps on the Net Charge

**Figure 1:**
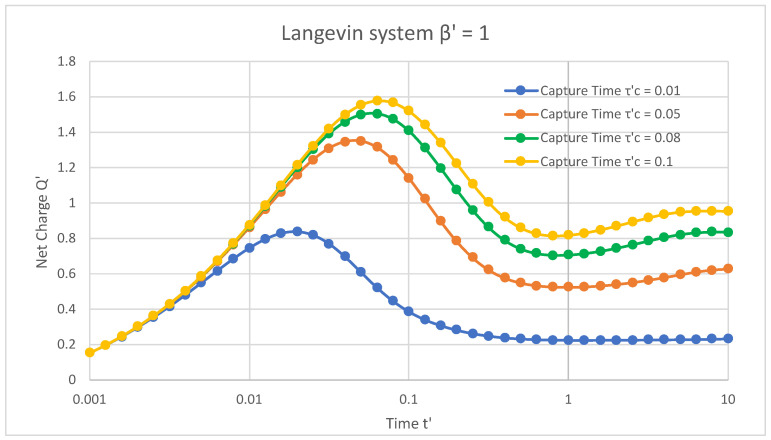
In the presence of traps and Langevin recombination of 1 between charges, the net charge varied with respect to time at a fixed release time of 0.6 and magnetic field of 1.

Figure 1 depicts the Langevin system in the presence of trap states, where the net normalised charge extracted from the device varies as a function of normalised time. The applied magnetic field and the release time of the trap states were fixed at 1 and 0.6, respectively, with changing capture times of trap states. For a capture time $\tau_{C}^{\prime }$ = 0.01, the net charge initially increased from 0.15 with an increase in time, and ranged from 0 to 0.019. However, it reached its maximum value of 0.83 for time 0.019 and then decreased and saturated at a value of 0.22 from time 0.025 to 10. For capture times $\tau_{C}^{\prime }$ = 0.05, 0.08, and 0.1, the net charge initially increased from 0.15 with an increase in time and then reached peak values of 1.35, 1.5, and 1.57 at times 0.05, 0.06, and 0.06, respectively. Furthermore, this net charge decreased with increasing times from 0.07 to 10 and saturated at values of 0.6, 0.8, and 0.9 for $\tau_{C}^{\prime }$ = 0.05, 0.08, and 0.1, respectively.

**Figure 2:**
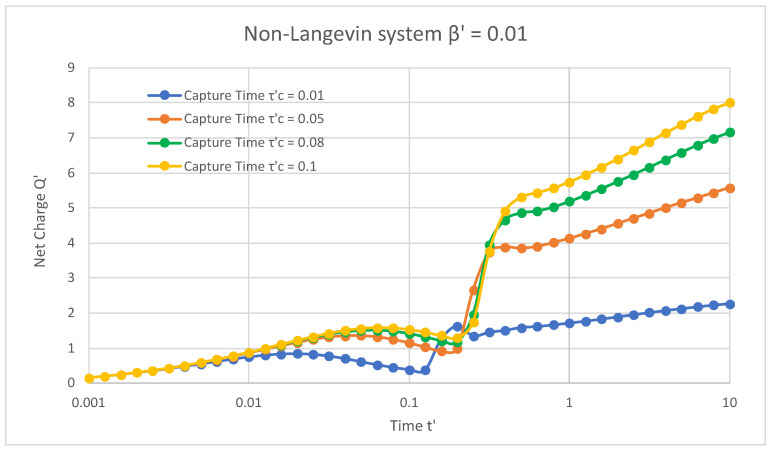
In the presence of traps and non-Langevin recombination of 0.01 between charges, the net charge varied with respect to time at a fixed release time of 0.6 and magnetic field of 1.

Figure 2 illustrates the non-Langevin system of 0.01 in the presence of trap states, where the net normalised charge extracted from the semiconductor device varied as a function of normalised time. The values of 1 and 0.6 were fixed for the applied magnetic field and the release time of the trap states, respectively, with varying capture times of the trap states. For a capture time $\tau_{C}^{\prime }$ = 0.01, the net charge initially increased from 0.15 with an increase in time and ranged from 0 to 0.019. However, it reached its highest value of 0.83 at time 0.019, then decreased to 0.37 from time 0.025 to 0.12, again increased to a maximum value of 1.62 at time 0.19, immediately dropped to 1.34 at time 0.25, and then finally increased and attained a maximum of 2.2 for an increasing time from 0.31 to 10. Similarly, for the capture times $\tau_{C}^{\prime }$ = 0.05, 0.08, and 0.1, it was observed that the net charge initially increased from 0.15 with an increase in time and then reached maximal values of 1.35, 1.5, and 1.58 at times 0.05, 0.06, and 0.06, respectively. Furthermore, this net charge decreased to 0.91, 1.16, and 1.28 with increasing times from 0.07 to 0.19 and then finally increased and arrived at values of 5.5, 7.1, and 8.0 with rising times of 0.25 to 10 for $\tau_{C}^{\prime }$ = 0.05, 0.08, and 0.1, respectively.

**Figure 3:**
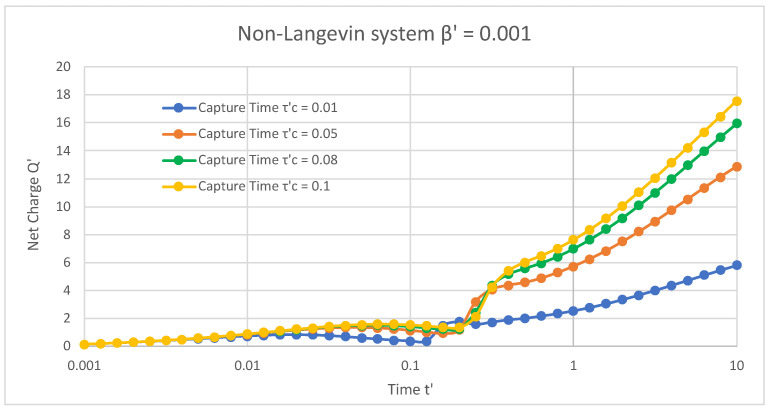
In the presence of traps and non-Langevin recombination of 0.001 between charges, the net charge varied with respect to time at a fixed release time of 0.6 and magnetic field of 1.

Figure 3 demonstrates the non-Langevin system of 0.001 in the presence of trap states, where the net normalised charge extracted from the semiconductor device varied with respect to normalised time. The release time of the trap states and the applied magnetic field were fixed at 0.6 and 1, respectively, with variations in the capture times of the trap states. For a capture time $\tau_{C}^{\prime }$ = 0.01, the net charge initially rose from 0.15 with an increase in time and ranged from 0 to 0.019. However, it attained its highest value of 0.83 at time 0.019, then reduced to 0.38 from time 0.025 to 0.12, again increased to the highest value of 1.76 at time 0.19, immediately dropped to 1.57 at time 0.25, and then finally increased and gained a maximum of 5.8 for the rising time from 0.31 to 10. Analogously, for the capture times $\tau_{C}^{\prime }$ = 0.05, 0.08, and 0.1, it was found that the net charge initially grew from 0.15 with a rise in time and then attained peak values of 1.35, 1.5, and 1.58 at times 0.05, 0.06, and 0.06, respectively. Moreover, this net charge dropped to 0.92, 1.21, and 1.34 with rising times of about 0.07 to 0.19, and then eventually increased and arrived at values of 12.87, 15.94, and 17.56 with rising times from 0.25 to 10 for $\tau_{C}^{\prime }$ = 0.05, 0.08, and 0.1, respectively.

Figure 1, Figure 2 and Figure 3 reveal that, in the presence of traps, increasing the capture time increased the net charge of the semiconductor device in both Langevin and non-Langevin systems. Moreover, the net charge extracted from the semiconductor device was about four and eight times greater in the case of the non-Langevin system (0.01 and 0.001, respectively) compared to the Langevin system, with an increase in the capture time at a fixed release time and magnetic field.

### 3.2. Implications of the Magnetic Field on Net Charge

**Figure 4:**
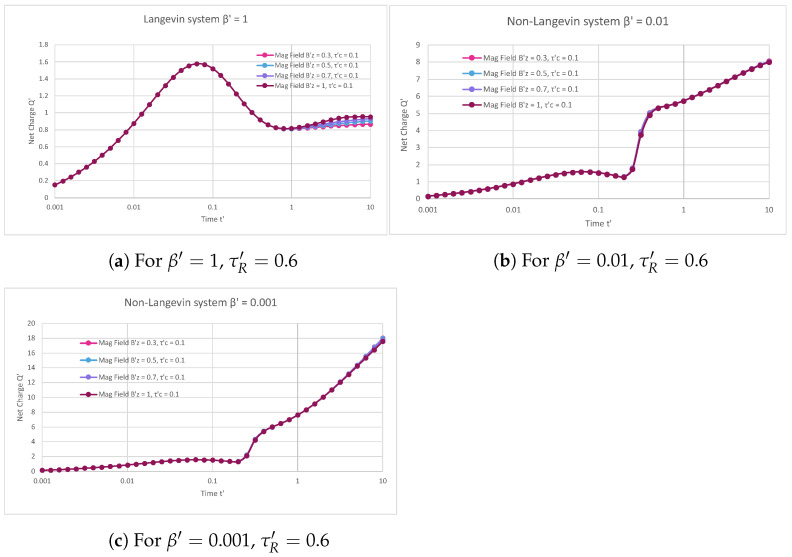
Net charge varying with respect to time for different magnetic fields at fixed capture and release times of 0.1 and 0.6, respectively, for Langevin system (**a**) and Non-Langevin system at values of (**b**) 0.01 and (**c**) 0.001.

Figure 4a indicates the Langevin system in the presence of trap states, where the net normalised charge extracted from the device varied as a function of normalised time. The capture and release times of the trap states were fixed at 0.1 and 0.6, respectively, with changing magnetic fields. For an applied magnetic field of 0.3, the net charge initially increased from 0.15 with an increase in time, and it ranged from 0 to 0.06. However, it reached its maximum value of 1.57 for time 0.06 and then decreased and saturated at a value of 0.86 from time 0.07 to 10. For the magnetic fields of 0.5, 0.7, and 1, the net charge initially increased from 0.15 with an increase in time and then reached a peak value of 1.57 at time 0.06. Furthermore, this net charge decreased with increasing times from 0.07 to 10 and saturated at values of 0.90, 0.93, and 0.95 for the magnetic fields of 0.5, 0.7, and 1, respectively. Moreover, it was observed that different magnetic field plots overlapped each other, with the highest peak value at 1.57 at time 0.06. Consequently, rising magnetic fields had little effect on the charge carriers, as they slightly increased the total charge of the semiconductor device.

Figure 4b describes the non-Langevin system of 0.01 in the presence of trap states, where the net normalised charge extracted from the semiconductor device varied as a function of normalised time. The values of 0.1 and 0.6 were fixed for the capture and release times of the trap states, respectively, with varying magnetic fields. In the case of an applied magnetic field of 0.3, the net charge initially increased from 0.15 with an increase in time and ranged from 0 to 0.06. However, it reached its highest value of 1.58 at time 0.06, decreased to 1.29 from time 0.07 to 0.19, and then finally increased and attained a maximum of 8.05 for an increasing time from 0.25 to 10. Similarly, for magnetic fields of 0.5, 0.7, and 1, it was observed that the net charge initially increased from 0.15 with an increase in time and then reached a maximal value of 1.58 at time 0.06. Further, this net charge decreased to 1.29 with increasing times from 0.07 to 0.19, and then finally increased and arrived at values of 8.03, 8.05, and 8.0 with rising times of 0.25 to 10 for magnetic fields of 0.5, 0.7, and 1, respectively. Additionally, it was observed that different magnetic field plots overlapped each other, with the highest maximal value at 1.58 at time 0.06. Therefore, increasing magnetic fields had a minor effect on the charge carriers, since they slightly altered the net charge of the semiconductor device.

Figure 4c represents the non-Langevin system of 0.001 in the presence of trap states, where the net normalised charge extracted from the semiconductor device varied with respect to normalised time. The capture and release times of the trap states were kept constant at 0.1 and 0.6, respectively, with variations in the applied magnetic field. With an applied magnetic field of 0.3, the net charge initially rose from 0.15 with an increase in time and ranged from 0 to 0.06. However, it attained its highest value of 1.58 at time 0.06, reduced to 1.35 from time 0.07 to 0.19, and then gradually increased and gained a maximum of 18.03 for the rising time from 0.25 to 10. For the applied magnetic fields of 0.5, 0.7, and 1, it was found that the net charge initially grew from 0.15 with a rising time and then attained a peak value of 1.58 at time 0.06. Moreover, this net charge dropped to 1.35 with rising times of about 0.07 to 0.19 and then eventually increased and arrived at values of 17.97, 17.82, and 17.56 with rising times from 0.25 to 10 for magnetic fields of 0.5, 0.7, and 1, respectively. In addition to this, it was observed that different magnetic field plots overlapped each other, with the highest peak value at 1.58 at time 0.06, which then reduced to 1.35 from time 0.07 to 0.19. Hence, the rising magnetic fields had a lesser impact on the charge carriers, because they slightly modified the net charge of the semiconductor device.

Figure 4a–c show that, in the presence of traps, increasing magnetic fields slightly increased the net charge of the semiconductor device in both the Langevin and non-Langevin systems. Also, these results indicate that, since the charge carriers were trapped in the trap states and no charge carriers were moving freely within the semiconductor device, the magnetic field had very little impact on the charge carriers in the presence of traps. Additionally, the net charge extracted from the semiconductor device was nearly four and eight times greater in the case of the non-Langevin system (0.01 and 0.001, respectively) compared to the Langevin system with rising magnetic fields at fixed capture and release times of the trap states.

## 4. Conclusions

The presence of traps due to defects or impurities in semiconductors can produce significant changes in the net charge of the semiconductor device. The results highlight the influence of magnetic fields and the capture and release times of trap states on the charge transport properties of organic semiconductors. When magnetic fields were varied at constant capture and release times of the trap states, it was observed that magnetic fields had less impact on the charge carriers, because the carriers were trapped within the trap states. On the other hand, by increasing the capture time of the trap states at a constant magnetic field and fixed release time, the net charge extracted from the semiconductor device increased with increasing capture time. Also, the net charge extracted from the semiconductor device was nearly four and eight times greater in the case of the non-Langevin system (0.01 and 0.001, respectively) when compared to the Langevin system. In conclusion, these results have important implications for analysing charge transport properties for the design and optimisation of organic semiconductor devices, and the device’s performance can be more accurately estimated and optimised, thereby leading to improved efficiency and reliability in accordance with the increasing demands of modern semiconductor technology.

## Data Availability

Not applicable.
